# The Modern Slavery Core Outcome Set: A Survivor-Driven Consensus on Priority Outcomes for Recovery, Wellbeing, and Reintegration

**DOI:** 10.1177/15248380231211955

**Published:** 2023-11-22

**Authors:** Sohail Jannesari, Bee Damara, Rachel Witkin, Cornelius Katona, Queenie Sit, Minh Dang, Jeanet Joseph, Emma Howarth, Olivia Triantafillou, Claire Powell, Sabah Rafique, Anitta Sritharan, Nicola Wright, Sian Oram, Sharli Anne Paphitis

**Affiliations:** 1 King’s College London, UK; 2 Brighton and Sussex Medical School, UK; 3 Survivor Alliance, London, UK; 4 Independent Consultant, UK; 5 The Helen Bamber Foundation, London, UK; 6 University of East London, UK; 7 University College London, UK; 8 University of Nottingham, UK

**Keywords:** modern slavery, human trafficking, outcomes, recovery, reintegration, survivor

## Abstract

There is no consensus on the outcomes needed for the recovery and reintegration of survivors of modern slavery and human trafficking. We developed the Modern Slavery Core Outcome Set (MSCOS) to address this gap. We conducted three English-language reviews on the intervention outcomes sought or experienced by adult survivors: a qualitative systematic review (4 databases, 18 eligible papers, thematic analysis), a rapid review of quantitative intervention studies (four databases, eight eligible papers, content analysis) and a gray literature review (2 databases, 21 websites, a call for evidence, 13 eligible papers, content analysis). We further extracted outcomes from 36 pre-existing interview transcripts with survivors, and seven interviews with survivors from underrepresented groups. We narrowed down outcomes via a consensus process involving: a three-stage E-Delphi survey (191 respondents); and a final consensus workshop (46 participants). We generated 398 outcomes from our 3 reviews, and 843 outcomes from interviews. By removing conceptual and literal duplicates, we reduced this to a longlist of 72 outcomes spanning 10 different domains. The E-Delphi produced a 14-outcome shortlist for the consensus workshop, where 7 final outcomes were chosen. Final outcomes were: “long-term consistent support,” “secure and suitable housing,” “safety from any trafficker or other abuser,” “access to medical treatment,” “finding purpose in life and self-actualisation,” “access to education,” and “compassionate, trauma-informed services.” The MSCOS provides outcomes that are accepted by a wide range of stakeholders and that should be measured in intervention evaluation.

## Background

An estimated 49.6 million people worldwide live in modern slavery (International Labour Organization & Walk Free Initiative of the Minderoo Foundation, 2022), which encompasses the “severe exploitation of other people for personal or commercial gain,” including human trafficking, forced labor and debt bondage (Anti-Slavery International, 2021). Survivors of modern slavery experience serious and long-term health, social, and economic consequences (Evans et al., 2022; Ottisova et al., 2016). However, high-quality evidence is lacking about how policies and services can effectively intervene to support survivor recovery, wellbeing, and reintegration (Dell et al., 2017).

The term “modern slavery” is a controversial one. It has been criticized as undermining international cooperation, trivializing historical slavery and being appropriated for political purposes (Dottridge, 2017; Faulkner, 2017). O’Connell Davidson (2016) has argued that the term risks equating today’s exploited persons to “things” rather than recognizing their agency and diverse experiences. However, charities such as Freedom United defend the use of the term “modern slavery” by highlighting its resonance with the public and power to galvanize global action (Ewart-James & Howard, 2017). Relatedly, Plant (2014) suggests that, while terminologies may vary, the essential focus remains on eradicating severe forms of exploitation. For the purposes of this research, we use “modern slavery” with an understanding of its limitations, but also its strategic value in generating a common understanding and facilitating discussions on the recovery and reintegration of survivors.

A major barrier to evaluating the effectiveness of support interventions—as well as to the synthesis and application of what evidence exists—is the high degree of variability of outcomes used in modern slavery research (Doherty et al., 2016; Graham et al., 2019; Wright et al., 2021). In their systematic review, for example, Graham et al. identified more than 25 broadly defined constructs measured across 53 studies (Graham et al., 2019). Fewer than half of the studies reported the measures used to collect information and the majority used study-specific measures, including items developed by the study research teams and individual items selected from multiple scales. Comparing the effectiveness of interventions, and ensuring quality, requires that the measurement of outcomes be standardized and consistently reported. Building consensus about which outcomes should be measured is therefore vital.

A further problem is the lack of survivor involvement characterizing many of the policies, programs, and evaluations that aim to support survivor recovery, with intervention outcomes rarely chosen—or even informed—by survivors. The outcomes targeted for improvement and measured for evaluations may therefore not be meaningful to their recovery and reintegration experiences. Nor is it clear to what extent outcomes reflect the concepts of success held by those that deliver or commission interventions. Existing academic research is largely focused on survivors’ physical and mental health, with limited consideration of other aspects of wellbeing (e.g., coping, social support), outcomes probably relevant to recovery (e.g., family relationships, employment, engagement in education or training, needs related to legal issues or advocacy), or service engagement (Graham et al., 2019).

The Core Outcome Measures for Effectiveness Initiative (COMET) (Williamson et al., 2017), which promotes the development of core outcome sets, provides a model for the development of a survivor-driven consensus on priority outcomes for supporting recovery after modern slavery. Core outcome sets are “agreed, standardized sets of outcomes” developed using consensus methods to identify and agree outcomes important to all stakeholders, and intended to be measured and reported across as a minimum in all clinical trials in specific areas of health or health care (COMET Initiative, 2020). Although initially intended for use in clinical trials and to support the monitoring and evaluation of health challenges, the concept can be extended to problems that require complex and multi-sectoral responses (Powell et al., 2022) as well as intervention evaluation, intervention design, and service delivery.

We developed a Modern Slavery Core Outcome Set (MSCOS) to support the future design and evaluation of interventions to support survivors of modern slavery. We conceptualized survivor health as broadly as possible, being open to the potential importance of social and educational outcomes. Similarly, we attempted to approach the concept of health interventions creatively, for example, allowing for human trafficking interventions that campaign for political change or raise awareness where relevant. We defined outcomes as “the direct or indirect result of a planned action that is facilitated by an outside party or program to facilitate survivor recovery, well-being and reintegration.” To ensure that the MSCOS was produced through a survivor-driven consensus process and that the outcomes were meaningful to survivors, we adopted a participatory approach to its development.

## Methods

**Figure 1.**
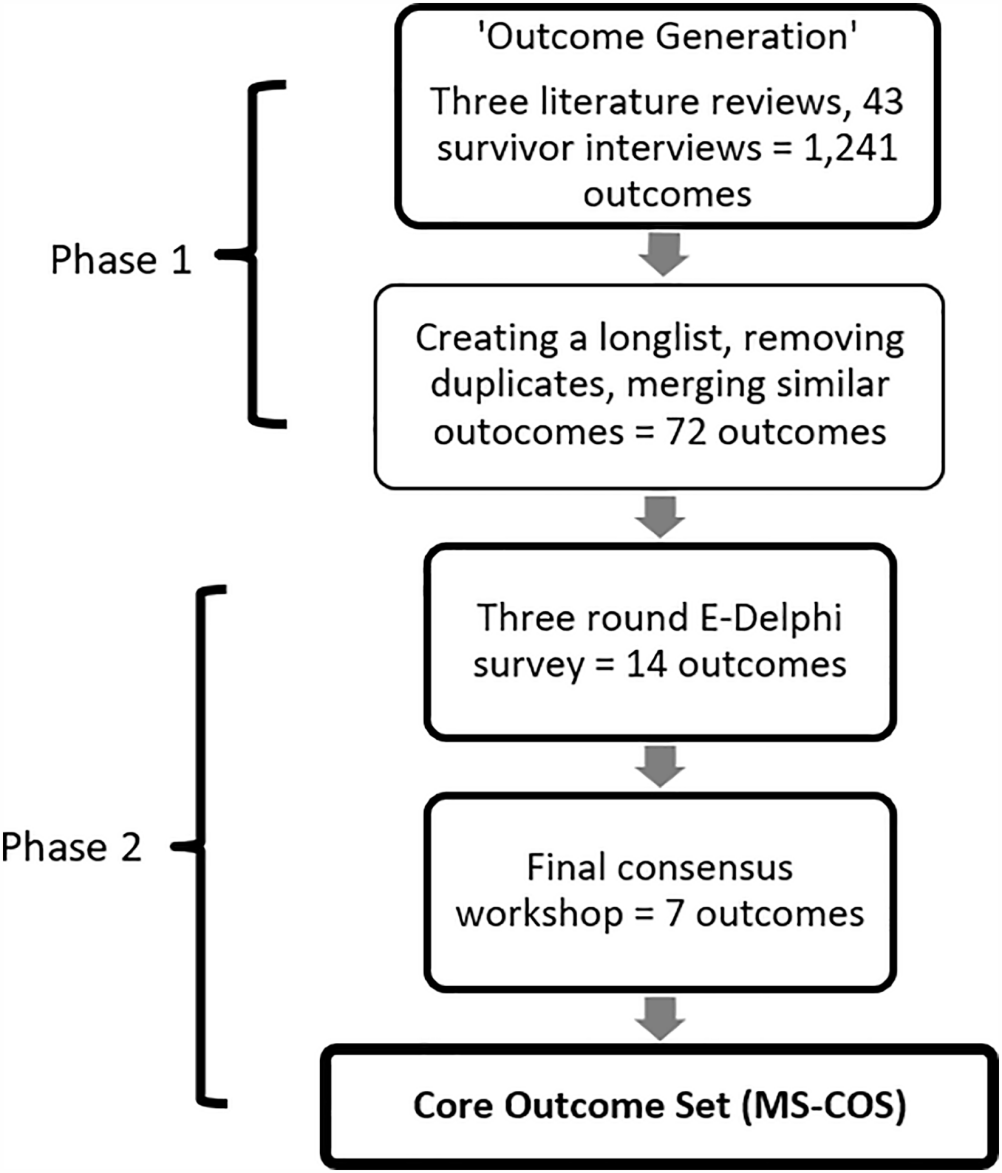
Overview of the Modern Slavery Core Outcome Set development process.

The study has been registered with the COMET initiative (ref 2317, https://www.comet-initiative.org/Studies/Details/2317) and adheres to the Core Outcome Set Standards for Reporting (Supplemental Appendix D). The protocol was not prospectively published. In line with COMET methodology (Williamson et al., 2017), a two-stage approach was used (see Figure 1). Phase 1 was generative in nature and, drawing upon literature reviews and qualitative interviews, produced a long list of candidate outcomes. In phase 2, stakeholder workshops and a three-stage e-Delphi were used to refine and gain consensus on the outcomes to be included in the core outcome set. We received ethical approval from the KCL Health Faculties Research Ethics Committee (HR/DP-21/22-26450 and HR/DP-21/22-26029).

### Survivor Involvement

We adopted a participatory research approach in this project. Participatory research can involve participant or lived experience participation in the research design, data collection, analysis, and dissemination. It exists on a spectrum from participants acting as advisors to being peer researchers. Adopting a participatory approach is one way of meeting growing calls for survivor leadership and involvement in trafficking research (e.g., Dang & Leyden, 2021). Survivor involvement can make research and services more effective, meaningful, and counter trafficking experiences of exploitation. Researchers working with people seeking sanctuary, a population that includes modern slavery survivors, have suggested that a participatory approach may be able to reduce harmful power dynamics between researchers and participants and disrupt some of the colonial power dynamics inherent in research and intervention practices (Jannesari, 2022). A participatory approach makes space for participant input, experience, and control and should produce more meaningful and relevant outcomes for our MSCOS.

The participatory approach adopted in this study was informed by the principles detailed in the Survivors Voices Charter (Perot et al., 2018), and strived in particular, to create “intentional space for dialog with survivors. . . [where] projects, events and research findings [have] survivors’ voices as a key input, allowing them to be the ‘experts by experience.’” Survivor Alliance, a survivor-led international network providing leadership training, consultancy, and research opportunities to survivors of modern slavery and human trafficking, was a partner organization to the research, supporting the inclusion of survivors throughout the research as well as the use of accessible and survivor-informed data collection and dissemination practices.

Peer researchers with experience of modern slavery were recruited through Survivor Alliance and embedded in the core project team at month three. Researchers worked with the academic team to design and deliver core project activities including the reviews, workshops, and E-Delphi. Researchers received training and support through the “Placing Survivor Voice and Wellbeing on the Policy and Evidence Map” program (University of Birmingham, 2022), as well as project-specific research training and support facilitated by the academic team. The establishment of a seven-member survivor research advisory board (RAB) was again facilitated by our partnership with Survivor Alliance and the RAB met bi-monthly to provide additional lived-experience guidance for the design and implementation of the project and the interpretation of findings. Finally, and as detailed below, survivors were involved as participants in the research. At all stages of the project, survivors were paid for their time according to NIHR (2022) participation guidelines.

### Participants

We recruited survivors, practitioners, academics, and policymakers for the stakeholder and consensus workshops and e-Delphi surveys (phase 2). We additionally recruited survivors to qualitative interviews (phase 1).

a. Survivors. We recruited survivors through survivor organizations including but not limited to Survivor Alliance. Experiences of modern slavery could have been either as adults or as children and did not have to have occurred in the UK. Survivors had to have accessed services or interventions at some point in their lives to be eligible to take part. Participation in the workshops and supplementary interviews was limited to English-speaking adults. Through international NGOs in India and South Africa, we also recruited survivors who spoke Hausa and Hindi to the e-Delphi; NGOs translated the questionnaires for non-English speaking participants.
b. Practitioners. We recruited adult, English speaking staff members and volunteers at any charity or not-for-profit organization working with survivors of modern slavery and human trafficking. We also recruited healthcare staff who worked with or had previously worked with survivors, or worked in a role relating to survivors such as in safeguarding or overseas charging. Practitioners from any country setting were eligible.
c. Academics. We recruited adult, English-speaking academics and students who had written or contributed to literature on modern slavery either through an academic paper, news article, or charity report. Academics from any country setting were eligible.
d. Policymakers: We recruited adult, English-speaking staff working for government departments, government-affiliated bodies (e.g., the UK Independent Anti-Slavery Commissioner’s Office), and public bodies (e.g., the UK National Health Service). They had to have a policy brief or job description that included modern slavery. In this category, we also included current/former MPs whose work was relevant to modern slavery and human trafficking, as well as NGO lobbyists. Policymakers from any country setting were eligible.

### Phase 1 Information Sources

Candidate outcomes were identified through (a) a series of three literature reviews; (b) secondary analysis of qualitative interview transcripts; (c) supplementary qualitative interviews.

#### Literature Reviews

Three literature reviews were conducted: (a) A review of peer-reviewed qualitative studies (led by SR). This review aimed to identify service and intervention outcomes sought or experienced by survivors of human trafficking and modern slavery. (b) A review of peer-reviewed intervention studies (led by AS). This review aimed to identify outcomes measured in studies evaluating interventions for survivors of human trafficking and modern slavery. The review proceeded in two steps, firstly identifying relevant systematic reviews and secondly screening individual studies included within those reviews. (c) A review of gray literature (led by SJ). This review aimed to identify intervention outcomes for survivors of human trafficking and modern slavery from reports, service evaluations and other non-academic research.

#### Search Strategies

Full search strategies can be found in Supplemental Appendix A. For the qualitative review, SR searched EMBASE, MEDLINE, HMIC, and PsycINFO using a combination of Medical Subject Headings (MeSH) and text words for the period January 1, 2000 to March 30, 2021. Further relevant qualitative studies were identified by expert recommendation through callouts via networks such as HEAL trafficking and VITA. For the interventions review, SO searched EMBASE, MEDLINE, HMIC, and PsycINFO using a combination of MeSH and text words for the period January 1, 2011 to July 2, 2021.

For the gray literature review, SJ searched the NICE Evidence Search and Open Gray databases and the websites of national and international anti-trafficking charities, survivor-led organizations, government websites in English-speaking countries (e.g., the United Kingdom, the United States and Australia), and bodies that sit between universities, charities, and governments. Searches were conducted between September 12, 2021 and December 26, 2021, with a lower date limit of September 12, 2021. Search terms were used where websites had search functionality, otherwise a manual review of the relevant sections of the websites (e.g., resources or reports sections) was undertaken. Further relevant reports were identified through requests to our project partners Survivor Alliance and Helen Bamber Foundation, the RAB, and workshop attendees and by review of records returned in but excluded from the qualitative and quantitative reviews as gray literature. Forwards and backwards citation tracking was conducted for all three reviews using Google Scholar and reference list screening, respectively.

#### Inclusion and Exclusion Criteria

Common across the three reviews was a focus on the outcomes of interventions and services received or sought by adult survivors of modern slavery. We defined modern slavery as the severe exploitation of other people for personal or commercial gain, including human trafficking, forced labor and debt bondage (Anti-Slavery International, 2021) and survivors as people who have lived experience of and have exited modern slavery (Survivor Alliance, 2022). We excluded studies whose main population was people who experienced modern slavery as a result of state forced labor, forced marriage or descent-based slavery as these categories typically sit under different policy areas than the categories we included. In studies with mixed ages, at least 75% of the sample were required to be aged 18 or over for the study or report to be included. Interventions of interest were psychosocial or psychological (studies reporting on pharmacological interventions were ineligible) and could operate at the individual, group, or community level. Outcomes of interest were those that related broadly to recovery and reintegration, including (a) health and wellbeing; (b) recovery; (c) functioning; (d) adversity and material deprivation; (e) safety and risk; (f) access to or experiences of resources, services, or programs; (g) education and employment; (h) legal status; (i) family and other interpersonal relationships. We required that papers and reports be published in English due to limitations in the languages spoken by the review team, but no restrictions were placed on country setting.

We additionally applied review-specific criteria. Studies were eligible for inclusion in the qualitative review if they: presented the results of peer-reviewed qualitative or mixed-methods research (including more than two lines of participant quotes); were published since 2000; included the perspectives of adult survivors of modern slavery; and reported primary accounts of experienced or expected outcomes of post-trafficking service provision. The interventions review first identified peer-reviewed systematic reviews for which the scope included intervention studies (either controlled or uncontrolled and with or without randomization) or program or service evaluations for adult survivors of modern slavery. Systematic reviews were required to have a structured search strategy which included electronic database searches and to have been published since 2011 (we judged that individual studies included in earlier reviews should also be included in comprehensive reviews published after this date). Individual studies reported in reviews meeting these criteria were then screened, and were eligible if they included evaluation of a defined activity, intervention, program, or service and at least one outcome was measured at the level of the intervention recipient or their family. Eligible for inclusion in the gray literature review were non-peer reviewed reports that included research of any design describing the outcomes of interventions and evaluations of services for adult survivors of modern slavery. We accepted reports published by governments, intergovernmental agencies, charities and other non-profit groups, and private companies. Research published in academic journals, book chapters, conference papers, theses, and dissertations were excluded, as was any material published solely by universities or within charity annual reports, and opinion pieces, blogs, articles, reports or other material that was not based on research.

#### Screening and Data Extraction

For all reviews, titles and abstracts were screened against the inclusion and exclusion criteria. Full-text screening was then conducted on eligible papers. A second reviewer reviewed a percentage of articles (25% in the qualitative review, 10% in the quantitative and gray literature review) independently in both phases. Disagreements between reviewers were resolved by discussion with a senior member of the MSCOS team (SO or SP). Data from the included studies and reports were extracted into an MS Excel spreadsheet. In addition to extracting information on outcomes, we extracted information on study design, study setting, sample size and characteristics (e.g., proportion of men and women, age, types of exploitation experienced, secure/insecure immigration status), intervention type, and methods of data collection and analysis.

#### Synthesis

Content analysis was used to extract outcomes from the intervention and gray literature reviews. Data from the qualitative review were analyzed using meta-ethnography (Atkins et al., 2008; Noblit and Hare, 1988), with first-order constructs (participant quotes), and second-order constructs (which included author interpretations) extracted and coded before synthesis of third-order constructs using an iterative process of reciprocal translation.

### Survivor Interviews

To identify additional outcomes of interest to survivors of modern slavery, we undertook a secondary analysis of anonymized interview transcripts from Wright et al. (2020) and conducted new supplementary interviews with adult survivors of modern slavery. Methods and sample characteristics are described in full elsewhere (Wright et al., 2020). Transcripts were shared following a data sharing agreement and content analysis used to extract outcomes relating to recovery and reintegration. One researcher with lived experience of modern slavery (BD) conducted seven semi-structured qualitative interviews with adult survivors of modern slavery between November 29, 2021 and June 1, 2022. Recruitment was facilitated by Survivor Alliance and our Survivor RAB, and purposive sampling used with the aim of interviewing individuals from groups underrepresented in the literature: UK, Albanian, Eritrean, and Vietnamese nationals; male survivors of labor exploitation for criminal activity; and individuals who do not access formal government-funded support for survivors of modern slavery. Participants gave written informed consent prior to interview and could withdraw at any stage. Interviews lasted between 30 and 90 min and followed trauma-informed principles (University of Buffalo, 2022). Topics covered included important recovery outcomes, milestones, achievements, and desires; differences between these and recovery outcomes participants perceived services as prioritizing, and what outcomes participants wanted services to prioritize. The topic guide was reviewed and approved by our RAB. Interviews were recorded and transcribed by BD, and content analysis used to extract outcomes. Sample characteristics are not provided to protect participant anonymity.

### Synthesis and Longlist Development

An outcome longlist was created by synthesizing the outcomes extracted from the interviews and literature reviews. Outcomes were placed in an Excel file with a name, description/indicator, the quote/passage of text they were extracted from, and a domain(s). Domains were drawn from the typology presented in Jannesari et al.’s review of social environmental factors associated with asylum seeker mental health and the social determinants of health described by the World Health Organization (Jannesari et al., 2020; WHO, 2022). Outcomes in the same domains that described similar concepts were merged by the research team. Where the same outcome was in more than one domain, the research team decided on which domain it was most relevant based on its description and similarity to other domain outcomes. The longlist formed the basis of the e-Delphi surveys and consensus workshops in phase 2. This longlist was reviewed by our Survivor RAB who suggested more positive framings for outcomes that emphasized survivor agency and aspirations for a better life. These outcomes moved away from the deficit framings common in health settings. For example, “coping with mental health problems” was subsumed within the outcomes of “self-compassion” and “acceptance.”

### Consensus Process

We aimed to establish a deliberative consensus (Haug, 2015) rather than a unanimous agreement on outcomes. Accordingly, though a wide range of stakeholders influenced the MSCOS, everyone agreed that the views of survivors should be centered.

#### Stakeholder Workshops

Prior to the e-Delphi survey, we held two invitation-only, half-day online workshops to build relationships with and introduce the project to key stakeholders (including survivors of modern slavery) and help us think about how to describe the outcomes in the e-Delphi. Both sessions were independently facilitated and included a debriefing space for survivors run by the Survivor Alliance. They were not part of data collection, therefore no findings from the workshops are presented.

#### E-Delphi Surveys

In phase 2, we used a three round, three panel e-Delphi to reduce and refine the longlist of outcomes developed in phase 1. We drew on the national and international professional networks of the research team and of the Modern Slavery Policy Evidence Center, as well as our Survivor RAB to recruit to the survey. Additionally, we contacted the authors of studies included in the phase 1 literature reviews and used snowball sampling. Participants were sorted into three groups: (a) academics; (b) practitioners/policy makers; and (c) survivors.

We built our E-Delphi survey using Qualtrics (www.qualtrics.com) software. Participants could complete the survey via computer, tablet, or mobile phone. Visually impaired participants were provided with a PDF version of the survey and supported by a member of the research team to complete it. This represented the length of time each round of the e-Delphi was open for. After this time, data was analyzed and used to inform the next round so could not be withdrawn. Data from participants who did not complete their survey were also withdrawn.

In the first survey, participants were presented with the outcomes longlist, organized by domain. They could comment upon or suggest changes to the included outcomes and suggest new outcomes for inclusion. Where two or more respondents (this minimum number was decided so that changes reflected some form of consensus) made a similar suggestion about the same outcome, outcomes descriptions were adjusted, new outcomes added, and original outcomes merged as appropriate. In instances where respondents expressed opposing opinions, the MSCOS research team discussed how to proceed.

In the second survey, participants received a revised outcomes longlist, which was again organized by domain. Participants were asked to rate each item for inclusion in the MSCOS on a five-point Likert scale (strongly agree, agree, neither agree nor disagree, disagree, and strongly disagree) and to choose their top five domains and outcomes for inclusion. Respondents also had the option to comment on why they had made these choices. Outcomes were ranked by subtracting the number of “strongly agrees” from the number of “neither agree nor disagrees.” Any outcome ranked below the median, that had been commented upon negatively, or was in an unpopular domain (defined as a domain in the bottom half of the rankings) was considered for removal. However, if an outcome was ranked in the top five by three or more participants, it was retained at this stage.

In the third and final survey, participants received a revised outcomes list. Outcomes were sorted randomly rather than by domain, reflecting the reduced number of outcomes. Respondents were asked to rate items for inclusion in the final MSCOS on a five-point Likert scale but could select the top-score (“strongly agree”) a maximum of 12 times. Again, there was space for respondent comments. The 12 outcomes that received the highest overall rankings plus additional items scoring within the survivor panel’s top 12 were included in the final consensus workshop.

#### Consensus Workshop

The consensus workshop was used to decide on the final outcomes for inclusion in the MSCOS. Participants were recruited from previous stages of the research. This workshop was facilitated by an independent facilitator, had a series of break out room discussions facilitated by MSCOS research team members, and adopted survivor-informed practices.

Prior to the workshop, participants were asked to rank their top and bottom three outcomes. During the workshop, participants discussed their rankings in small groups, following these discussions they individually ranked their top three outcomes through an anonymous online Google Form. The results of the overall participant rankings and survivor participant rankings were shared with all workshop participants in a collective discussion before participants returned to small groups to continue their discussions. After this final discussion, participants were asked to reselect their top three outcomes. The final MSCOS included the six outcomes that received the most votes overall plus any outcomes that were in the survivor top six but not in the overall top six. Descriptors for each of these outcomes were developed with survivors. All outcome descriptors include qualitative indicators with the potential for development of implementable standards. These qualitative indicators and descriptors can feasibly be expressed as a potential set of standards that could be further developed to be measured quantitatively, qualitatively, and through survivor self-report.

## Results

### Phase 1

Across the multiple activities contributing to phase 1—three literature reviews and interviews with 41 survivors of modern slavery—we identified 1,241 candidate outcomes. The reviews included 39 papers (interventions review 8 papers, qualitative literature review 18 papers, gray literature review 13 papers) reporting on 1,335 participants (interventions review 240 participants, qualitative literature review 214 participants, gray literature review 881 participants), and contributed 398 outcomes (interventions review 33 outcomes, qualitative literature review 35 outcomes, gray literature review 330 outcomes). Studies were predominantly conducted in North America and Europe and focused mainly on trafficking for sexual exploitation. Information on the studies included in each of the reviews is presented in Supplemental Appendix B. Analysis of interview transcripts identified 843 outcomes. These included 584 outcomes from our secondary analysis of 36 interview transcripts (an average of 16 per interview) and 259 outcomes from interviews conducted with 7 survivors identified as being in groups previously underrepresented in the literature (an average of 37 per interview). This initial list of 1,241 outcomes was reduced to a 72-item longlist, organized into ten domains.

#### Review of Intervention Studies

Mental health outcomes (e.g., depression, PTSD) featured heavily in the review of intervention studies: four of the eight included studies used structured instruments to screen for or diagnose mental health problems (George et al., 2010; Munsey et al., 2018; Ostrovschi et al., 2011; Robjant et al., 2017). Two studies (Cerny et al., 2019; Mangum et al., 2019) used a structured instrument to assess performance of daily activities like self-care, leisure, and productivity, and two used measures developed by NGOs that encompassed a wide range of outcomes, including housing, social health, employment, and legal/immigration issues (Potocky, 2010; Shareck et al., 2020).

#### Review of Qualitative Studies

The review of qualitative studies generated outcomes organized under four themes. The first, “facets of service provision” identified the resources, activities, and psychological support needed for post-trafficking support, and focused on preparing for a life beyond immediate aftercare, while “personal desired outcomes from aftercare provision” described outcomes desired by survivors including independence and agency, stability, greater self-efficacy, formation of an identity and safety. The third theme, “qualities displayed by service providers” highlighted the importance of non-judgmental, compassionate, empowering approaches and authenticity from services, explaining that for many survivors, working with compassionate staff was an outcome in itself. Finally, “recommendations for services” emphasized the need for aftercare provision to provide holistic, specialized, and long-term care and support.

#### Review of Gray Literature

The review of gray literature produced a large number of outcomes covering a range of areas, but particularly prominent were those relating to services (e.g., services keeping their promises). wellbeing (e.g., being loved), and survivor agency (e.g., amplifying survivor voices, being heard, taking the lead [in services], not being treated like a victim). Outcomes related to peer support (e.g., connecting to other survivors, starting peer support groups) also emerged as important.

#### Qualitative Interviews

A third of the outcomes extracted from our secondary analysis of interview transcripts focused on mental health and wellbeing (e.g., positivity, self-esteem, self-awareness, anxiety, suicidality); unsurprising, given that interviews focused on mental health recovery. A fifth of extracted outcomes related to feeling “normal” and being able to function and participate in everyday society (e.g., feeling human, feeling heard, being able to sleep, being social, being able to use public transport). Outcomes relating to immigration status were fewer in number but were described as being of great importance to mental health. Analysis of the seven supplementary interviews added nuance to and drew links between previously extracted outcomes. For example, they illustrated the intimate link between safety and housing, revealing serious incidents of violence and continued abuse in managed/provider accommodation.

#### Longlist Development

The initial list of 1,241 outcomes was reduced to a 72-item longlist through discussion within the research team and reflection on feedback from the initial stakeholder workshop and RAB (including, e.g., to frame outcomes more positively than was often the case in the literature) These outcomes were organized into ten domains: (a) creating change; (b) supportive services; (c) rights, justice and dignity; (d) health and wellbeing; (e) safety; (f) agency and purpose; (g) belonging and social support; (h) opportunities; (i) recognition, understanding and awareness; and (j) consistency and stability.

### Phase 2

Sample information and aggregate results for all three e-Delphi surveys are provided in Supplemental Appendix C. The sample was majority female and United Kingdom-based, although the proportion of male and international participants increased across the three rounds.

We recruited 53 participants to the first e-Delphi survey and allocated them to three panels: survivors (*n* = 53), academics (*n* = 9), and policymakers and practitioners (*n* = 8). Twenty-three changes were made to the outcomes longlist because of the first e-Delphi survey: 16 outcomes were renamed (e.g., “cherishing the everyday” was altered to “reclaiming normalcy and appreciating the everyday”), three outcomes were added (e.g., affordable and reliable transportation), and four outcomes were merged or eliminated. A list of the outcomes considered can be found on the MSCOS (2022) website.

An amended longlist of 72 outcomes was considered by 64 stakeholders (*n* = 43 survivors, *n* = 8 academics, and *n* = 13 policymakers and practitioners) in the second e-Delphi survey, 48 (75%) of whom had also completed the previous round. Following analysis, 10 outcomes were merged into four new outcomes (e.g., “housing stability and independence” and “secure and protected housing” were merged into “secure and suitable housing,” while “timely and sustained psychological support” was subsumed within “long-term consistent support”) and 30 eliminated. Also merged were the two least popular domains, “creating change” and “agency and purpose.” They became simply “agency and purpose.”

Round 2 was the final round of the E-Delphi survey and had the most respondents at 74 (*n* = 39 survivors, *n* = 12 academics, and *n* = 23 policy makers and practitioners). Of these, 74% had completed the previous round. Respondents were asked to rank 38 outcomes. Following analysis of survey findings, the twelve outcomes that received the highest overall rankings were selected for consideration at the final consensus workshop. Two outcomes—“survival needs and state support” and “finding purpose in life and self-actualization”—were also taken forward as these were ranked within the top twelve of the survivors’ panel (see Supplemental Appendix C for full results).

The final consensus workshop was attended by 46 participants, 38 of whom identified as survivors. Following voting, the 6 outcomes that received the highest overall scores and were included in the MSCOS were: (a) long-term consistent support; (b) secure and suitable housing; (c) safety from any trafficker or other abuser; (d) access to medical treatment; (e) access to education; and (f) compassionate, trauma-informed services. A seventh outcome—(g) finding purpose in life and self-actualization—was among the highest scoring outcomes for survivors and was also included in the MSCOS (see Table 1).

**Table 1.** Critical Findings from the Review, Interviews, and Consensus Process.

Final outcomes included in the Modern Slavery Core Outcome Set	
Long-term consistent support	Support services should be advocated for at the right time and available when they are required in accordance with each survivor’s individual circumstances. It is important that survivors can access support that is long-term (e.g., therapeutic care and individual support specifically tailored to each person’s assessed needs, risks, and circumstances). Assessment of needs and risks should be revisited and updated on a regular basis and services available for as long as is required. A key outcome feature is that support is consistent, and it enables survivors to build a trusting relationship with professionals. It is important that support staff have training and pastoral supervision so that they do not suffer professional burnout and can continue to provide the long-term consistent support that is needed.
Secure and suitable housing	Survivors should live in a place they can call home, where they feel safe and secure, can exercise freedom and independence, and live without suffering, abuse, or exploitation. Housing should offer private personal space, be hygienic, have enough peace to be able to rest and sleep, and preclude worries about being evicted. Key outcome features include safe house accommodation being gender-sensitive, allowing for the proper investigation of complaints, having cooking and cleaning facilities, not being overcrowded, and being a place where survivors feel respected.
Safety from any trafficker or other abuser	This outcome includes a safe rescue process as well as sustained safety from all traffickers and abusers. It is critical that survivors live free from fear that perpetrators will recapture them, find out where they live, or threaten them in some way. Safety from new perpetrators who can target victims for re-trafficking or harm them in other ways is also vital. Ongoing safety can involve multiple aspects such as: having a landline to call emergency services in a safe house; living far from traffickers and their associates; and the police being careful in the way they handle cases. This outcome includes psychological safety from traffickers.
Access to medical treatment	This outcome is about ensuring that survivors have access to adequate services to meet their health needs. This includes having access to dental treatment. It requires, for example, having sufficient funds for transport to attend appointments and funding for therapy if this is not freely available. It also includes being registered with a GP and it could include access to culturally appropriate support. There is a desperate need for therapists to specialize in evidence-based trauma therapy to help survivors. Specific group therapies should exist for survivors to complement individual therapy.
Access to education	Key features include access to appropriate educational institutions and the availability of free courses and colleges; not being discriminated against by educational institutions in terms of course applications and eligibility; and sufficient funds to travel for courses and legal permission to study (sometimes denied by immigration laws). Access to education also includes foundational courses for work preparedness as well as less formal learning, such as being able to learn and practice new skills for example, IT, sewing and crafts, photography, art and design, etc.
Compassionate, trauma-informed services	This outcome describes the need for staff who are trained and experienced in working with survivors who have traumatic histories. Survivors need to be able to trust all the professionals who work with them including police, immigration authorities, support workers, social workers, and shelter staff. This means developing trusting relationships, working to realistic expectations, supporting survivors to understand all the information they are being given, communicating to survivors in their language, and being honest. At a very basic level, this outcome is about staff treating survivors as human beings, listening to their stories and needs, and being a positive force in people’s lives. All services need to be as inclusive and sensitively delivered as possible.
Finding purpose in life and self-actualization	This outcome is about a feeling of optimism and fulfillment. The idea of being able to have hope to dream and desire to live is crucial, as is being able to tolerate good and bad days without fully losing this sense of hope. A key outcome feature is self- actualization understood as the ability to follow passions in life and living life to the fullest. This could include, for example, using talents, setting goals for self- advancement, and articulating personal goals and dreams.

## Discussion

We utilized consensus methods and a survivor-driven process to create a MSCOS. The set comprises seven outcomes that should be measured as standard in future modern slavery research on interventions for survivor recovery and reintegration. These outcomes also provide a framework for policy and service design and evaluation. We have outlined the practice, policy, and research implications of our research in Table 2 and discuss these further in the MSCOS report (Paphitis & Jannesari, 2023).

**Table 2.** Practice, Policy, and Research Implications.

1. Researchers, practitioners, and policymakers should use the Modern Slavery Core Outcome Set (MSCOS) to think about interventions more broadly. This means considering all MSCOS outcomes in intervention development and evaluation.
2. Where an intervention doesn’t cover all MSCOS outcomes, researchers, practitioners, and/or policymakers should either consider amending the intervention or partnering with services and interventions that do.
3. Stakeholders should consider how outcomes can work on many different levels, including the individual, organizational, governmental, and societal levels, and the importance of structural factors when designing and evaluating interventions.
4. When working with individual-level outcomes, practitioners and researchers should be careful not to disproportionately burden survivors. They should consider setting self-development goals and work targets for other stakeholders.
5. All MSCOS outcomes should be measured at consistent, regular time points regardless of someone’s circumstances or time since their experience of trafficking.
6. Survivors should be involved in and remunerated for conducting research and NGO activities, with roles that reflect people’s different experiences and life circumstances. As part of this, survivors should be offered comprehensive induction as well as mental health support for the project duration.

It is important to note that the core outcomes should be used as a set, and we encourage researchers and practitioners to consider all seven outcomes simultaneously. Viewing the core outcomes as a set is critical since the outcomes included span a variety of domains that are often addressed separately across interventions or services, leading to a lack of integration in provision. In approaching the outcomes as a set, we aim to encourage researchers and services providers to adopt complex and multi-level approaches to designing services and interventions (recognizing that this will often require improving cross-sector and inter-agency collaboration).

The MSCOS is not an exhaustive set, and researchers and practitioners should recognize that further outcomes can and should be used to respond to specific experiences of survivors and adapt intervention designs and evaluations to the local context and to survivor demographics. The results of this study have enabled us to compile a longlist of 38 additional potential outcomes that can be used to supplement the MSCOS, which is detailed in the MSCOS report (Paphitis & Jannesari, 2023).

For this project, we defined outcomes as the “direct or indirect results of planned actions facilitated by an outside party or program with the aim of facilitating survivor recovery, wellbeing and integration post-trafficking.” This definition covers a wide range of actions from various stakeholders. Accordingly, our MSCOS is both multi-level and holistic, including outcomes across domains that have traditionally been addressed separately in interventions. The outcomes are not limited to survivor outcomes alone, but any outcomes that might serve survivors and impact their recovery, wellbeing, and reintegration. Survivors cannot be solely responsible for their recovery and reintegration: institutions and systems must also play a crucial role.

While some interventions already use a wide range of outcomes (Potocky’s, 2010) evaluation of the Florida Freedom Partnership, for instance, looked at 43 outcomes in areas across “housing, food, immigration status, mental health, health. . . education and employment status, and life skills”), we acknowledge that service providers may face capacity limitations. Thus, we emphasize the importance of multi-agency working in providing long-term, high-quality support, as highlighted by Hemmings et al.’s (2016) review of survivor health needs: the importance of multi-agency working in providing long-term, quality support. Encouragingly, Such et al.’s (2020) recent review of 17 studies identified several examples of cross-sectoral, multi-agency approaches in survivor service provision.

We acknowledge that some of the outcomes included in the MSCOS may be viewed as planned actions that lead to outcomes rather than outcomes themselves. However, we believe that each of the outcomes is a crucial result in the journey of survivors toward recovery and reintegration. They were identified as key life goals and symbols of reintegration into mainstream society, or the result of reclaiming some of what was lost during the trafficking process.

To prioritize the views of survivors and follow the principles of epistemic and actional deference (Pearlman & Elizabeth, 2022), we aimed to create a deliberative consensus (Haug, 2015) in which survivors’ views were centered and weighted more heavily. Although we ensured that a broad range of stakeholders had the opportunity to contribute and influence the development of the MSCOS, we believe that it is both morally and epistemically necessarily to “believe the testimony of people about ‘harms that relate to their [marginalised] identity’” (Pearlman & Elizabeth, 2022). Some outcomes included in the MSCOS reflect long-established findings, such as the fact that survivors continue to face insecure and dangerous living conditions after exiting trafficking, which negatively impacts health and wellbeing (Kiss et al., 2015), or that survivors experience multiple barriers to accessing healthcare even when being supported by specialist post-trafficking charities (Westwood et al., 2016). Other outcomes have been less commonly reported, perhaps reflecting this project’s inclusion of voices not previously represented in the literature and the strength of survivor involvement. For example, “finding purpose in life and self-actualisation” was rarely discussed in the literature, except for in a doctoral thesis on a “holistic work intervention program for women survivors” in South Africa (Sambo, 2019), in which service providers discussed the therapeutic importance supporting survivors to find purpose and self-actualize. Our MSCOS reflects the most crucial issues that survivors are facing now. We note that the consensus position can change in response to major global events in the trafficking sector and could become redundant if the MSCOS is widely accepted and implemented.

### Strengths and Limitations

The project followed established methodology for the development of core outcome sets, and the resulting MSCOS was informed by the views of several hundred survivors, practitioners, policymakers, researchers, and other stakeholders. A key strength of the project was its survivor-driven approach, which resulted in a higher-quality core outcome set and research process. The involvement of survivor advisors and peer researchers led to the identification of outcomes that were overlooked by other members of the team. The RAB provided guidance that improved the accessibility of workshop and survey materials, and led to the involvement of a greater number and wider range of survivors.

While advisory group members and peer researchers described positive impacts of their involvement (see Damara et al. forthcoming), one peer researcher dropped out due to external pressures unrelated to the project. In hindsight, external supervision with a trauma-informed specialist may have helped her manage her concerns. The project would have also benefited from a longer induction period for survivors, in which we could have discussed individual strengths and areas of contribution.

The project’s partnership between universities, charities, and survivor organizations, along with our extensive professional networks, supported the recruitment of participants and the identification of gray literature. However, there were also limitations. The literature reviews and workshops were conducted in English only, leading to a geographic skew. Additionally, there was a disproportionate focus in the literature on the experiences of people trafficked for sexual versus labor exploitation. The project attempted to address this gap through our supplementary qualitative interviews and the consensus process.

#### Next Steps

We have established a community of practice to support the utilization and further development of the MSCOS, including by gaining consensus on developing standards, best practice, measures, and indicators. A community of practice can be defined as “a group of people who share a common concern, a set of problems, or an interest in a topic and who come together to fulfill both individual and group goals” (Community of Practice, 2023, p. 1).

The MSCOS Community of Practice (MSCOS, 2022) was established by RW and QS, is hosted by the Helen Bamber Foundation, and led by the RAB. Consistent guidance and input are provided by the MSCOS team. Through its website and newsletter, the Community of Practice (a) showcases practice, viewpoints and perspectives on MSCOS outcomes; (b) shares stakeholder reports, blogs, videos, events and publications; (c) details models and frameworks related to the MSCOS; and (d) hosts online discussion forums with RAB members on developing measurable MSCOS standards. It has 250 subscribers from government, survivor-led organizations and survivor leaders, civil society, and charity service providers, health service professionals, lawyers, police and victim navigators, and academics and students.

Although each MSCOS outcome has a detailed description that provides suggestions for potential indicators, additional effort is required to explore this in greater depth. Future research on measures and indicators will require a review of existing measures employed in modern slavery research and practice; work with survivors on the identified indicators and measures to evaluate which are the most suitable and least intrusive (and whether survivors prefer to self-report on certain items); and the creation of a rubric of standards to complement the MSCOS.

## Conclusion

The development and implementation of evidence-led support for survivors of modern slavery requires a consensus on the definition and measurement of recovery and reintegration outcomes. The MSCOS provides a minimum set of outcomes that should be reported across interventions aimed at supporting survivor recovery and reintegration. These outcomes have been identified as important to recovery and reintegration and prioritized by survivors, practitioners, academics, and policymakers. The study demonstrates that a fully participatory approach to core outcome set development can be taken. By being embedded into research and practice, the MSCOS can improve the quality, value, and relevance of modern slavery research and evaluation and, ultimately, improve outcomes for survivors.

## Supplemental Material

sj-docx-1-tva-10.1177_15248380231211955 – Supplemental material for The Modern Slavery Core Outcome Set: A Survivor-Driven Consensus on Priority Outcomes for Recovery, Wellbeing, and Reintegration Supplemental material, sj-docx-1-tva-10.1177_15248380231211955 for The Modern Slavery Core Outcome Set: A Survivor-Driven Consensus on Priority Outcomes for Recovery, Wellbeing, and Reintegration by Sohail Jannesari, Bee Damara, Rachel Witkin, Cornelius Katona, Queenie Sit, Minh Dang, Jeanet Joseph, Emma Howarth, Olivia Triantafillou, Claire Powell, Sabah Rafique, Anitta Sritharan, Nicola Wright, Sian Oram and Sharli Anne Paphitis in Trauma, Violence, & Abuse
