# Supplementary material for: The Modern Slavery Core Outcome Set: A Survivor-Driven Consensus on Priority Outcomes for Recovery, Wellbeing, and Reintegration
Source: Trauma Violence Abuse. 2023 Nov 22;25(3):2377–89. doi: 10.1177/15248380231211955 (PMC11155204; doi:10.1177/15248380231211955)
Supplement: sj-docx-1-tva-10.1177_15248380231211955 – Supplemental material for The Modern Slavery Core Outcome Set: A Survivor-Driven Consensus on Priority Outcomes for Recovery, Wellbeing, and Reintegration [file sj-docx-1-tva-10.1177_15248380231211955.docx]

**Appendix A**

**Search Strategy for rapid review of quantitative intervention studies**

The following electronic databases were searched from 2011: Medline, Embase, PsycInfo, CINAHL, and Web of Science. Searching included expert recommendations of relevant broader studies. The search strategy included MeSH terms relating to human trafficking and modern slavery. Key word terms for human trafficking/modern slavery and systematic reviews were used. Where the function is available, searches were limited to retrieve only systematic reviews. As part of the review involves collecting definitions of human trafficking/modern slavery and their operationalisation within intervention studies, any study deemed to fit within the umbrella by the research team was included.

***Search terms***

1. Human trafficking/

2. Enslaved Persons/

3. Enslavement/

4. Modern slavery.mp

5. Human trafficking.mp

6. (human OR women OR woman OR man OR men OR person OR people OR sex* OR victim OR survivor) adj2 (traffick*).mp

7. 1 OR 2 OR 3 OR 4 OR 5 OR 6

8. Systematic review/

9. Systematic review.mp

10. 8 OR 9

11. 7 AND 10

**Search Strategy for systematic review of qualitative studies**

Electronic databases including EMBASE, MEDLINE, HMIC and PsycINFO were used. Reference list screening and forward citation tracking using a combination of Medical Subject Headings (MeSH) and text words was conducted to search for additional relevant material. This were used with papers identified as eligible for the review after full text screening. In addition, other relevant qualitative studies or reviews were identified by expert recommendation. Networks such as ‘HEAL trafficking’, the VITA network and a Modern Slavery Research Google Group was used to do so.

**EMBASE Strategy**

***Trafficking terms.***

1. Human Trafficking/ (632)
2. Enslavement/ (285)
3. Enslaved Persons/ (333)
4. ((human* or wom?n or m?n or person* or people* or sex* or victim*) adj3 traffic*).mp. (3579)
5. slave*.mp. (3273)
6. enslave*.mp. (353)
7. [servitude.mp](https://eur03.safelinks.protection.outlook.com/?url=http%3A%2F%2Fservitude.mp%2F&data=04%7C01%7Csabah.rafique%40kcl.ac.uk%7Ccd82790f79ef4994a19308d8d04fc2a9%7C8370cf1416f34c16b83c724071654356%7C0%7C0%7C637488388272607976%7CUnknown%7CTWFpbGZsb3d8eyJWIjoiMC4wLjAwMDAiLCJQIjoiV2luMzIiLCJBTiI6Ik1haWwiLCJXVCI6Mn0%3D%7C1000&sdata=e2uNtibK43xt1K3dEC50%2BsXs7%2FDvZCjOGBre9F1ZcYM%3D&reserved=0). (86)
8. modern-slave*.mp. (44)
9. ((sex* or physic*) adj3 (exploit*)).mp. (1583)
10. ((forced) adj3 (work* or employ* or labo?r* or prostitution*)).mp. (739)
11. ((bonded or exploit*) adj3 (work* or employ* or job* or labo?r* or prostitution*)).mp. (1314)

Combined with ‘or’ (10225)

***Qualitative terms.***

*Q*ualitative analysis/ (65452)

1. Qualitative research/ (86137)
2. Exp interviews/ or exp interpretative phenomenological analysis/ or exp content analysis/ (315406)
3. (("semi-structured" or semistructured or unstructured or informal or "in-depth" or indepth or "face-to-face" or guide or guides) adj2 (interview* or discussion*)).mp. (123045)
4. (qualitative or "focus group" or ethnograph* or "key informant" or "participant-observation" or action-research" or action research" or "thematic analysis" or theme* or "content analysis" or "discourse analysis" or "narrative analysis" or "cooperative inquiry" or "appreciative inquiry" "grounded theory" or phenomenolog* or "convenience sample" or "purposive sample" or audiorecording or "audio recording").mp. (530426)

Combined with ‘or’ (754978)

Trafficking terms and qualitative terms combined with ‘and’ (782)

**MEDLINE Strategy**

***Trafficking terms.***

1. Human Trafficking/ (476)
2. Enslavement/ (126)
3. Enslaved Persons/ (61)
4. ((human* or wom?n or m?n or person* or people* or sex* or victim*) adj3 traffic*).mp. (2828)
5. slave*.mp. (2985)
6. enslave*.mp. (448)
7. [servitude.mp](https://eur03.safelinks.protection.outlook.com/?url=http%3A%2F%2Fservitude.mp%2F&data=04%7C01%7Csabah.rafique%40kcl.ac.uk%7Ccd82790f79ef4994a19308d8d04fc2a9%7C8370cf1416f34c16b83c724071654356%7C0%7C0%7C637488388272607976%7CUnknown%7CTWFpbGZsb3d8eyJWIjoiMC4wLjAwMDAiLCJQIjoiV2luMzIiLCJBTiI6Ik1haWwiLCJXVCI6Mn0%3D%7C1000&sdata=e2uNtibK43xt1K3dEC50%2BsXs7%2FDvZCjOGBre9F1ZcYM%3D&reserved=0). (91)
8. modern-slave*.mp. (46)
9. ((sex* or physic*) adj3 (exploit*)).mp. (1193)
10. ((forced) adj3 (work* or employ* or labo?r* or prostitution*)).mp. (620)
11. ((bonded or exploit*) adj3 (work* or employ* or job* or labo?r* or prostitution*)).mp. (1224)

Combined with ‘or’ (8707)

*Qualitative terms.*

1. qualitative research/ (60932)
2. Exp interview/ or exp interpretative phenomenological analysis/ or exp content analysis/ (29596)
3. (("semi-structured" or semistructured or unstructured or informal or "in-depth" or indepth or "face-to-face" or guide or guides) adj2 (interview* or discussion*)).mp. (91617)
4. (qualitative or "focus group" or ethnograph* or "key informant" or "participant-observation" or action-research" or action research" or "thematic analysis" or theme* or "content analysis" or "discourse analysis" or "narrative analysis" or "cooperative inquiry" or "appreciative inquiry" "grounded theory" or phenomenolog* or "convenience sample" or "purposive sample" or audiorecording or "audio recording").mp. (392369)

Combined with ‘or’ (453337)

 Trafficking terms and qualitative terms combined with ‘and’ (543)

**APA PsycInfo Strategy**

***Trafficking terms.***

1. Human Trafficking/ (1088)
2. Enslavement/ (0)
3. Enslaved Persons/ (0)
4. ((human* or wom?n or m?n or person* or people* or sex* or victim*) adj3 traffic*).mp. (1990)
5. slave*.mp. (2826)
6. enslave*.mp. (540)
7. [servitude.mp](https://eur03.safelinks.protection.outlook.com/?url=http%3A%2F%2Fservitude.mp%2F&data=04%7C01%7Csabah.rafique%40kcl.ac.uk%7Ccd82790f79ef4994a19308d8d04fc2a9%7C8370cf1416f34c16b83c724071654356%7C0%7C0%7C637488388272607976%7CUnknown%7CTWFpbGZsb3d8eyJWIjoiMC4wLjAwMDAiLCJQIjoiV2luMzIiLCJBTiI6Ik1haWwiLCJXVCI6Mn0%3D%7C1000&sdata=e2uNtibK43xt1K3dEC50%2BsXs7%2FDvZCjOGBre9F1ZcYM%3D&reserved=0). (215)
8. modern-slave*.mp. (42)
9. ((sex* or physic*) adj3 (exploit*)).mp. (1523)
10. ((forced) adj3 (work* or employ* or labo?r* or prostitution*)).mp. (496)
11. ((bonded or exploit*) adj3 (work* or employ* or job* or labo?r* or prostitution*)).mp. (695)

Combined with ‘or’ (7158)

***Qualitative terms.***

1. qualitative methods/ (9485)
2. qualitative measures/ (84)
3. Exp interviews/ or exp interpretative phenomenological analysis/ or exp content analysis/ (34718)
4. (("semi-structured" or semistructured or unstructured or informal or "in-depth" or indepth or "face-to-face" or guide or guides) adj2 (interview* or discussion*)).mp. (92229)
5. (qualitative or "focus group" or ethnograph* or "key informant" or "participant-observation" or action-research" or action research" or "thematic analysis" or theme* or "content analysis" or "discourse analysis" or "narrative analysis" or "cooperative inquiry" or "appreciative inquiry" "grounded theory" or phenomenolog* or "convenience sample" or "purposive sample" or audiorecording or "audio recording").mp. (364906)

Combined with ‘or’ (413277)

Trafficking terms and qualitative terms combined with ‘and’ (1248)

**HMIC Search Strategy**

***Trafficking terms.***

1. Human Trafficking/ (17)
2. Enslavement/ (0)
3. Enslaved Persons/ (0)
4. ((human* or wom?n or m?n or person* or people* or sex* or victim*) adj3 traffic*).mp. (53)
5. slave*.mp. (32)
6. enslave*.mp. (3)
7. [servitude.mp](https://eur03.safelinks.protection.outlook.com/?url=http%3A%2F%2Fservitude.mp%2F&data=04%7C01%7Csabah.rafique%40kcl.ac.uk%7Ccd82790f79ef4994a19308d8d04fc2a9%7C8370cf1416f34c16b83c724071654356%7C0%7C0%7C637488388272607976%7CUnknown%7CTWFpbGZsb3d8eyJWIjoiMC4wLjAwMDAiLCJQIjoiV2luMzIiLCJBTiI6Ik1haWwiLCJXVCI6Mn0%3D%7C1000&sdata=e2uNtibK43xt1K3dEC50%2BsXs7%2FDvZCjOGBre9F1ZcYM%3D&reserved=0). (2)
8. modern-slave*.mp. (7)
9. ((sex* or physic*) adj3 (exploit*)).mp. (62)
10. (forced) adj3 (work* or employ* or labo?r* or prostitution*)).mp. (29)
11. ((bonded or exploit*) adj3 (work* or employ* or job* or labo?r* or prostitution*)).mp. (27)

Combined with ‘or’ (188)

***Qualitative terms.***

1. Qualitative analysis/ (80)
2. qualitative research/ (1298)
3. exp qualitative techniques/ (327)
4. Exp interviews/ or exp interpretative phenomenological analysis/ or exp content analysis/ (1167)
5. (("semi-structured" or semistructured or unstructured or informal or "in-depth" or indepth or "face-to-face" or guide or guides) adj2 (interview* or discussion*)).mp. (5506)
6. (qualitative or "focus group" or ethnograph* or "key informant" or "participant-observation" or action-research" or action research" or "thematic analysis" or theme* or "content analysis" or "discourse analysis" or "narrative analysis" or "cooperative inquiry" or "appreciative inquiry" "grounded theory" or phenomenolog* or "convenience sample" or "purposive sample" or audiorecording or "audio recording").mp. (15764)

Combined with ‘or’ (18550)

Trafficking terms and qualitative terms combined with ‘and’ (14)

**Search Strategy for grey literature review**

Search terms varied depending on the resource being searched. Some websites had no or limited search functionality. In these cases, a resources/reports/research part of the website was searched and manually looked through. Where search functionality was available, a combination of the following terms was used: modern slavery, human trafficking, trafficked, sexual exploitation, forced labour, debt bondage, bonded labour, intervention, evaluation, recovery, outcomes. The depth of search depended on the depth of the database.

1. Grey databases: NICE Evidence Search, Open Grey

2. Records in the qualitative review and umbrella review marked as grey literature.

3. Websites of UK-focussed charities (conducting the majority of their work in the UK, with offices in the UK) with a dedicated modern slavery and trafficking focus: Freedom Fund, Hestia, Salvation Army, Snowdrop Foundation, British Red Cross, Helen Bamber Foundation, Human Trafficking Foundation, After Exploitation, African Rainbow Family, Focus on Labour Exploitation, Kalayaan, Unseen, Refuge, Anti-Slavery International (and the Anti-Trafficking Monitoring Group), Medaille Trust, Labour Behind the Label, Anti-Trafficking and Labour Exploitation Unit, Freedom from Torture.

4. Survivor led organisations: The International Survivors of Trafficking Advisory Board (no website - email), The Voice of Domestic Workers, Survivor Alliance, Filipino Domestic Workers Association (no website – email).

5. Foreign and international charity websites (conducting the majority of their work in abroad, with offices abroad): Freedom Fund, La Strada International, International Organisation for Migration, International Labour Organisation, Global Alliance Against Trafficking in Women, Walk Free Foundation, Polaris Project, the AIRE Centre.

6. UK government websites: Home Office, Department for International Development, Independent Anti-Slavery Commissioner, Care Quality Commission.

7. Foreign English-speaking governments: US Department of Justice, US Department of Health and Human Services, Australian Department of Social Services, Australian Department of Home Affairs, Australian Department of Community Safety and Multicultural Affairs.

8. Bodies that sit between universities, NGOs and governments: University of Nottingham Rights Lab, University of Toledo Human Trafficking and Social Justice Institute.

9. Call for evidence across all these stakeholders.

**Appendix B**

**Table 1**

*Summary of Review of Interventions Studies*

| **Lead Author** | **Study Country** | **Year** | **Sample** | **Outcomes assessed** |
| --- | --- | --- | --- | --- |
| George | United States | 2020 | 5 men and 31 women (mainly experience of sex trafficking) | Physical and mental health (e.g., PTSD, hepatitis C, pelvic pain, depression) |
| Shareck | Canada | 2020 | 100 – 120 women est. (all experience of sex trafficking) | Health, addiction, housing, legal issues, social support networks, education and employment. |
| Cerny | United States | 2019 | 8 women (all experience of sex trafficking) | Meaningful activities, task behaviours (e.g., decision making, problem-solving), executive functioning skills (e.g., planning, initiating), occupational performance. |
| Magnum | United States | 2019 | 15 women (all experience of sex trafficking) | Sensory modulation (e.g., self-regulation of emotions, self-esteem, resilience), basic functions (e.g., cooking, using the telephone) executive functions, occupational performance. |
| Munsey | United States | 2018 | 11 women (all experience of sex trafficking) | Depression, PTSD and self-esteem |
| Robjant | United Kingdom | 2017 | 10 women (all experience of sex trafficking) | PTSD, distress |
| Ostrovschi | Moldova | 2011 | 120 women (mainly experience of sex trafficking) | Psychiatric diagnosis (e.g., PTSD, depression) |
| Potocky | United States | 2010 | 6 undocumented migrant men and 37 women (experience of sex and labour trafficking) | Shelter/food, immigration issues, mental health, social and emotional health, English language ability, and employment/education. |

**Table 2**

*Summary of Review of Qualitative Studies*

| **Lead Author** | **Year** | **Country** | **Sample Size** | **M** | **F** | **Nationalities and Ethnicities** | **Exploitation Type(s)** |
| --- | --- | --- | --- | --- | --- | --- | --- |
| Castaner | 2021 | United States | 14 | 0 | 14 | Mexican, Central American | Sex trafficking |
| Mumey | 2021 | United States | 6 | 0 | 6 | African American, Arab American, Latinx | Sex trafficking |
|  |  |  |  |  |  |  |  |
| Balfour | 2020 | Ghana | 27 | 0 | 27 | Ghanaian | Domestic/Labour |
| Da Silva | 2019 | India | 10 | 0 | 10 | Indian | Sex trafficking |
|  |  |  |  |  |  |  |  |
| Doyle | 2019 | Ireland | 15 | 2 | 13 | Pakistani, South African, Indian, Filipino, Kenyan, Nigerian, Malawian | Labour |
| Evans | 2019 | United States | 15 | 0 | 15 | Hispanic, Caucasian, African American, Dutch Canadian, Native American | Sex trafficking |
| Hodge | 2019 | United States | 21 | 21 | 0 | Latin American, Asian | Labour and sex trafficking |
|  |  |  |  |  |  |  |  |
| Orme | 2019 | United States | 12 | 0 | 12 | Hispanic, Caucasian | Sex trafficking |
|  |  |  |  |  |  |  |  |
| Viergever | 2019 | Netherlands | 14 | 5 | 9 | African, Eastern European, Asian, Middle Eastern | Sex trafficking |
|  |  |  |  |  |  |  |  |
| Hopper | 2018 | United States | 17 | 0 | 17 | African American, Caucasian, Hispanic | Sex trafficking |
| Bruijn | 2017 | United States | 8 | 0 | 8 | Caucasian, African American | Sex trafficking |
|  |  |  |  |  |  |  |  |
| Eldridge | 2017 | United States | 9 | 0 | 9 | Caucasian, Hispanic | Sex trafficking |
|  |  |  |  |  |  |  |  |
| Rajaram | 2016 | United States | 22 | 0 | 22 | Caucasian, African American, Hispanic | Sex trafficking |
| Dahal | 2015 | Nepal | 10 | 0 | 10 | Nepalese | Sex trafficking |
| McCrory | 2015 | United States | 6 | 0 | 6 | African American, Caucasian, Hispanic, Asian | Sex trafficking |
|  |  |  |  |  |  |  |  |
| Jones | 2014 | United States | 8 | 0 | 8 | Caucasian, African American, Caribbean, Romanian | Sex trafficking |
| Busch-Armendariz | 2011 | United States | 9 | 0 | 9 | Unspecified | Labour and sex trafficking |
| Westebbe | 2004 | Thailand | 5 | 0 | 5 | Thai | Labour and sex trafficking |

**Table 3**

*Summary of Review of Grey Literature*

| **Title** | **Year** | **(n)** | **Organisation(s)** | **Country** |
| --- | --- | --- | --- | --- |
| Access to legal advice and representation for survivors of modern slavery | 2021 | 30 | Modern slavery and Human Rights Policy and Evidence Centre | UK |
| Underground Lives: Male Victims of Modern Slavery | 2021 | 42 | Hestia | UK |
| Going places: Journeys to recovery | 2020 | 107 | Rights Lab | UK |
| The lived realities of sustained liberation in Uttar Pradesh and Bihar, India: an evaluation of survivor experiences | 2020 | 88 | Rights Lab | India |
| Study of HHS Programs Serving Human Trafficking Victims | 2019 | 341 | US Department of Health and Human Services | United States |
| Dignity, Not Destitution | 2019 | 21 | Kalayaan | UK |
| Pro-Act UK Pilot Report | 2018 | n/a | Focus on Labour Exploitation | UK |
| Report on the contribution of the NCATS to the identification and assistance for trafficking victims | 2017 | n/a | USAID, IOM, NRCVT, Different and Equal, Vatra Centre | Albania |
|  |  |  |  |  |
| Day 46 | 2016 | 31 | Human Trafficking Foundation | UK |
| Conversations of Empowerment | 2015 | 14 | Survivor Alliance | Global |
| Evaluation of the effectiveness of measures for the integration of Trafficked persons | 2013 | 112 | IOM | Belgium, France, Hungary, Italy, UK |
|  |  |  |  |  |
| The Impact of the Republic of Moldova Anti-Trafficking Policy on the Trafficked Persons' Rights | 2013 | 30 | La Strada International | Moldova |
| Evaluation of Comprehensive Services for Victims of Human Trafficking: Key Findings and Lessons Learned | 2007 | 33* | U.S. Department of Justice | United States |
| Comprehensive Services for Survivors of Human Trafficking: Findings from Clients in Three Communities | 2006 | 34 | Urban Institute | United States |

*Note:* The survivor participants in this paper are the same as those from the US Department of Health and Human Services (2019) and were excluded

**Appendix C**

| **Table 1**  *E-Delphi Participant Characteristics* | | | |
| --- | --- | --- | --- |
| **Participant Variables** | **Round 0**  **N=53(%)** | **Round 1**  **N= 64(%)** | **Round 2**  **N= 74(%)** |
| **Stakeholder group** |  |  |  |
| Survivors | 36 (67.9) | 43 (67.2) | 39 (52.7) |
| Researchers/academics | 9 (17.0) | 8 (12.5) | 12 (16.2) |
| Service Providers | 4 (7.5) | 9 (14.1) | 9 (12.2) |
| Policy Makers | 4 (7.5) | 4 (6.3) | 14 (18.9) |
| **Demographics** |  |  |  |
| Man | 4 (7.5) | 10 (15.6) | 14 (18.9) |
| Woman | 47 (88.7) | 54 (84.4) | 60 (81.1) |
| Transgender | 1 (1.9) | 0 | 0 |
| Prefer not to say/did not report | 1 (1.9) | 0 | 0 |
| N self-declaring a disability | 5 | 10 | 9 |
| Median age in years (range) | 36 (18-68) | 38 (23-68) | 39 (23-89) |
| **Ethnicity** |  |  |  |
| Asian* | 18 (34.0) | 12 (18.8) | 17 (23.0) |
| African* | 9 (17.0) | 24 (37.5) | 21 (28.4) |
| White * | 18 (34.0) | 20 (31.3) | 27 (36.5) |
| Hispanic/Latino | 0 | 0 | 1 (1.4) |
| Mixed ethnic group | 0 | 2 (3.1) | 4 (5.4) |
| Did not self-describe* | 8 (15.1) | 6 (9.4) | 4 (5.4) |
| **Country of current location** |  |  |  |
| UK | 45 (84.9) | 46 (71.9) | 52 (70.3) |
| South Africa | 1 (1.9) | 4 (6.3) | 1 (1.4) |
| Nigeria | 1 (1.9) | 2 (3.1) | 1 (1.4) |
| USA | 5 (9.4) | 5 (7.8) | 8 (10.8) |
| Kenya | 1 (1.9) | 5 (7.8) | 2 (2.7) |
| Cameroon | 0 | 1 (1.6) | 1 (1.4) |
| India | 0 | 1 (1.6) | 5 (6.8) |
| Germany | 0 | 0 | 1 (1.4) |
| Israel | 0 | 0 | 1 (1.4) |
| Denmark | 0 | 0 | 1 (1.4) |
| Guernsey | 0 | 0 | 1 (1.4) |

*Note:* ‘African’ ethnic group includes Kikuyu, Igbo, ‘Asian’ ethnic group includes Filipino, Bangladeshi, Indian, Maharashtra, Tamil, Punjabi and Pakistani, ‘White’ ethnic group includes White British, European and other e.g., White Jewish and indigenous British, ‘Did not self-describe’ group includes all those who did not write down an identifiable ethnicity in the free-text box.

**Table 2**

*Changes to outcomes following the first stage of the E-Delphi (CN = changing names of outcomes MO = merging/moving of outcomes to different domains NO = new outcome EO = eliminated outcome)*

| **Outcome Domain** | **Outcomes** | **Change Type** | **Previous outcome name (if applicable)** |
| --- | --- | --- | --- |
| Consistency and Stability | Reclaiming normalcy and appreciating the everyday  Keeping busy  Life skills  Being financially responsible for self and others  Housing stability and independence  Long term, consistent support  Healthy lifestyle  Affordable and reliable transportation | CN              NO | ‘Cherishing the everyday’ |
| Recognition, Understanding and Awareness | Improved understanding of mental health treatments  Respect and recognition from healthcare practitioners and service providers  Belief and respect from public authorities, courts and tribunals    Knowledge of rights and entitlements  Living a stigma free life  Less public judgement, more understanding | CN    CN    CN    CN | Understanding of treatment’  Respect and recognition from practitioners’  ‘Belief and respect from immigration officials, police, judges and services’    ‘Resisting victimising stereotypes’ |
| Opportunities | Obtaining and maintaining meaningful employment  Career Progression  Personal and family prosperity  Self-expression and opportunity through host country language  Obtaining meaningful qualifications  Access to education | CN | ‘Obtaining employment’ |
| Belonging and Social Support | Feeling comfortable in social environment  Healthy relationships  Having people to talk to  Socialising  Living in an appropriate or desired location  Being part of a community | CN | ‘Living in a good location’ |
| Agency and Purpose | Finding purpose in life and self-actualization  Meaningful and creative activities  Self-sufficiency, control and independence  Reclaiming the past  Moving on and starting a new life  Becoming an advocate for self and giving to others | CN | ‘Becoming an advocate for self and others’ |
| Safety | Preventing re-exploitation  Safety from any trafficker or other abuser  A safe mental health service, work and home environment  Family safety and contact  Secure and protected housing | CN  CN | ‘Safety from trafficker’  ‘A safe mental health service and home environment’ |
| Health and Wellbeing | Celebrating and thinking positively  Self-compassion, acceptance and self-worth  Self-awareness and emotional expression  Processing trauma  Spiritual well-being  Improved physical wellbeing  Coping with mental health problems  Access to medical treatment  Timely and sustained psychological support  Being able to seek support | CN | ‘Improved vital functions’ |
| Rights, Justice and Dignity | No racism  No discrimination against LGBTQ+  Dignified treatment of survivors  Permission to work  Family reunification  Immigration status and documentation  Better immigration systems  Freedom of movement  Dignity in living conditions  Charity accountability    Survival needs and state support  Prosecutions    Access to quality legal representation | CN  NO  CN      CN        EO/MO      CN | ‘Less racism’    ‘Fair treatment of survivors’    ‘Immigration status’        See Domain: ‘Supportive Services’      ‘Compensation and prosecutions’ |
| Supportive Services | Service accountability  Compassionate, trauma-informed staff behaviour  Staff that fight for your rights  Being able to trust support workers and other practitioners  Quality, well-resourced support  Survivor choice in services  Inclusive and sensitive support | NO    EO  CN | Change from ‘worker’ to ‘workers’ |
| Creating Change | Grappling with and tackling oppression  Solidarity and being part of a movement  Amplifying survivor voices and creating change  Improving policy  Recognition of activism  Survivor leadership  Increased male involvement | CN | ‘Demanding new government policies’ |

**Table 3**

***E-Delphi second stage ranking of outcomes for exclusion (EO = eliminated outcome, MO = merging/moving of outcomes to different domains, a/b/c/d corresponds to outcomes that were combined together)***

| **Outcome** | **Rating** | **Change imposed (if any)** |
| --- | --- | --- |
| Safety from any trafficker or other abuser | 46 |  |
| Compassionate, trauma-informed staff behaviour | 43 | MO (a) |
| Long term, consistent support | 40 | MO (c) |
| Secure and protected housing | 40 |  |
| Access to education | 40 |  |
| Dignified treatment of survivors | 37 | MO (b) |
| Survival needs and state support | 37 |  |
| Access to medical treatment | 35 |  |
| Preventing re-exploitation | 35 |  |
| Knowledge of rights and entitlements | 35 |  |
| Processing trauma | 34 |  |
| Access to quality legal representation | 34 |  |
| No racism | 33 |  |
| Being able to trust support workers and other practitioners | 32 | MO (a) |
| Having people to talk to | 32 |  |
| Healthy relationships | 31 |  |
| Belief and respect from public authorities, courts, and tribunals | 30 | MO (b) |
| Housing stability and independence | 29 |  |
| Timely and sustained psychological support | 29 | MO (c) |
| Better immigration systems | 29 |  |
| Quality, well-resourced support | 29 |  |
| Improving policy | 29 |  |
| Survivor leadership | 29 | MO (d) |
| Life skills | 28 |  |
| Dignity in living conditions | 28 |  |
| Survivor choice in services | 28 |  |
| Inclusive and sensitive support | 28 | MO (a) |
| Immigration status and documentation | 27 |  |
| Amplifying survivor voices and creating change | 27 | MO (d) |
| Being part of a community | 27 |  |
| Respect and recognition from healthcare practitioners and service providers | 27 | MO (b) |
| Reclaiming Normalcy and Appreciating the Everyday | 26 |  |
| Self-compassion, acceptance and self-worth | 26 |  |
| Moving on and starting a new life | 26 |  |
| A safe mental health service, work and home environment | 25 |  |
| Obtaining and maintaining meaningful employment | 25 |  |
| MEDIAN 24.5 |  |  |
| Coping with mental health problems | 24 |  |
| Being able to seek support | 24 |  |
| Service accountability | 24 |  |
| Finding purpose in life and self-actualisation | 24 |  |
| Permission to work | 22 | EO |
| Being financially responsible for self and others | 21 | EO |
| Advocating for self and giving to others | 21 | EO |
| Improved understanding of mental health treatments | 20 | EO |
| Less public judgement, more understanding | 20 | EO |
| Self-sufficiency, control and independence | 19 | EO |
| Obtaining meaningful qualifications | 18 | EO |
| Affordable and reliable transportation | 17 | EO |
| No discrimination against LGBTQ+ | 17 | EO |
| Freedom of movement | 17 | EO |
| Living in an appropriate or desired location | 17 | EO |
| Living a stigma free life | 17 | Retained (3 ‘top 5’ votes) |
| LOWER QUARTILE |  |  |
| Self-awareness and emotional expression | 16 | EO |
| Improved physical wellbeing | 16 | EO |
| Prosecutions | 16 | EO |
| Recognition of activism | 16 | EO |
| Meaningful and creative activities | 16 | EO |
| Career progression | 16 | EO |
| Family reunification | 14 | EO |
| Grappling with and tackling oppression | 14 | EO |
| Personal and family prosperity | 14 | EO |
| Self-expression and opportunity through host country language | 14 | EO |
| Celebrating and thinking positively | 13 | EO |
| Healthy lifestyle | 12 | EO |
| Feeling comfortable in social environment | 12 | EO |
| Socialising | 12 | Retained (3 ‘top 5’ votes) |
| Increased male involvement | 11 | EO |
| Keeping busy | 9 | EO |
| Solidarity and being part of a movement | 9 | EO |
| Family safety and contact | 9 | EO |
| Reclaiming the past | 6 | EO |
| Spiritual wellbeing | 4 | EO |

**Table 4**

*E-Delphi Round 2: top 12 ranked outcomes (all participants and survivors)*

|  | | | | | |
| --- | --- | --- | --- | --- | --- |
| **All participants, N = 76** | | | **Survivors, N = 39** | | |
| Outcome | Score | Ranking position | Outcome | Score | Ranking position |
| Safety from any trafficker or other abuser | 101 | 1 | Secure and suitable housing | 45 | 1 |
| Secure and suitable housing | 98 | 2 | Compassionate, trauma-informed services | 45 | 1 |
| Access to medical treatment | 87 | 3 | Access to education | 45 | 1 |
| Access to education | 86 | 4 | Access to quality legal representation | 43 | 2 |
| Access to quality legal representation | 86 | 4 | Access to medical treatment | 42 | 3 |
| Preventing re-exploitation | 85 | 5 | Knowledge of rights and entitlements | 42 | 3 |
| Compassionate, trauma-informed services | 85 | 5 | Safety from any trafficker or other abuser | 41 | 4 |
| Knowledge of rights and entitlements | 84 | 6 | Long term, consistent support | 39 | 5 |
| Obtaining and maintaining meaningful employment | 78 | 7 | Preventing re-exploitation | 38 | 6 |
| Long term, consistent support | 75 | 8 | Life skills | 38 | 6 |
| Life skills | 73 | 9 | Survival needs and state support | 38 | 6 |
| Coping with mental health problems | 73 | 9 | Coping with mental health problems | 38 | 6 |
|  |  |  | Finding purpose in life and self-actualisation | 38 | 6 |

**Appendix D**

**Core Outcome Set-STandards for Reporting: The COS-STAR Statement Checklist**

| **SECTION/TOPIC** | **ITEM No.** | **CHECKLIST ITEM** | **REPORTED ON PAGE NUMBER** |
| --- | --- | --- | --- |
| TITLE/ABSTRACT | | | |
| Title | 1a | Identify in the title that the paper reports the development of a COS | 1 |
| Abstract | 1b | Provide a structured summary | 1 |
| INTRODUCTION | | | |
| Background and Objectives | 2a | Describe the background and explain the rationale for developing the COS. | 2-3 |
|  | 2b | Describe the specific objectives with reference to developing a COS. | 3-4 |
| Scope | 3a | Describe the health condition(s) and population(s) covered by the COS. | 1-2 |
|  | 3b | Describe the intervention(s) covered by the COS. | 3-4 |
|  | 3c | Describe the setting(s) in which the COS is to be applied. | 3-4 |
| METHODS | | | |
| Protocol/Registry Entry | 4 | Indicate where the COS development protocol can be accessed, if available, and/or the study registration details. | 4 |
| Participants | 5 | Describe the rationale for stakeholder groups involved in the COS development process, eligibility criteria for participants from each group, and a description of how the individuals involved were identified. | 6-7 |
| Information Sources | 6a | Describe the information sources used to identify an initial list of outcomes. | 7-15 |
|  | 6b | Describe how outcomes were dropped/combined, with reasons (if applicable). | 7-15 |
| Consensus Process | 7 | Describe how the consensus process was undertaken. | 13-15 |
| Outcome Scoring | 8 | Describe how outcomes were scored and how scores were summarised. | 14 |
| Consensus Definition | 9a | Describe the consensus definition. | 13 |
|  | 9b | Describe the procedure for determining how outcomes were included or excluded from consideration during the consensus process. | 13-15 |
| Ethics and Consent | 10 | Provide a statement regarding the ethics and consent issues for the study. | 12, 14 |
| RESULTS | | | |
| Protocol Deviations | 11 | Describe any changes from the protocol (if applicable), with reasons, and describe what impact these changes have on the results. | n/a |
| Participants | 12 | Present data on the number and relevant characteristics of the people involved at all stages of COS development. | 18-19, Appendix B |
| Outcomes | 13a | List all outcomes considered at the start of the consensus process. | 19 |
|  | 13b | Describe any new outcomes introduced and any outcomes dropped, with reasons, during the consensus process. | 18-20, Appendix B |
| COS | 14 | List the outcomes in the final COS. | 19-20, Table 1 |
| DISCUSSION | | | |
| Limitations | 15 | Discuss any limitations in the COS development process. | 22-23 |
| Conclusions | 16 | Provide an interpretation of the final COS in the context of other evidence, and implications for future research. | 20-24 |
| OTHER INFORMATION | | | |
| Funding | 17 | Describe sources of funding/role of funders. | Title Page |
| Conflicts of Interest | 18 | Describe any conflicts of interest within the study team and how these were managed. | Title Page |

*From: Kirkham JJ, Gorst S, Altman DG, Blazeby JM, Clarke M, Devane D, et al. (2016) Core Outcome Set–STAndards for Reporting: The COS-STAR Statement. PLoS Med 13(10): e1002148. https://doi.org/10.1371/journal.pmed.1002148*
